# Tumor-Associated Neutrophils in Hepatocellular Carcinoma Pathogenesis, Prognosis, and Therapy

**DOI:** 10.3390/cancers13122899

**Published:** 2021-06-10

**Authors:** Konstantinos Arvanitakis, Ioannis Mitroulis, Georgios Germanidis

**Affiliations:** 1 First Department of Internal Medicine, AHEPA University Hospital, Aristotle University of Thessaloniki, 54636 Thessaloniki, Greece; kostarvanit@gmail.com; 2 Basic and Translational Research Unit, Special Unit for Biomedical Research and Education, School of Medicine, Faculty of Health Sciences, Aristotle University of Thessaloniki, 54636 Thessaloniki, Greece; 3 First Department of Internal Medicine, University Hospital of Alexandroupolis, Democritus University of Thrace, 68100 Alexandroupolis, Greece; imitroul@med.duth.gr

**Keywords:** tumor-associated neutrophils, hepatocellular carcinoma, tumorigenesis, neutrophil-to-lymphocyte ratio, treatment resistance

## Abstract

**Simple Summary:**

Hepatocellular carcinoma is the most prevalent primary liver cancer, accounting for >80% of primary liver cancers worldwide. Inflammation has come to light as a hallmark of cancer development, and it has become increasingly apparent over the past decade that tumor-associated inflammation drives the involvement of neutrophils in disease progression and metastasis. Infiltrating TANs exhibit either anti-tumorigenic (N1) or protumorigenic (N2) phenotypes. In the vast majority of solid human tumors, high infiltration with tumor-associated neutrophils (TANs) has been correlated with increased tumor growth, lymph node metastasis, and poor prognosis overall, whereas evidence from other studies advocate that under certain conditions, TANs exert cytotoxic and inhibitory activity towards tumor cells, halting the progression of cancer. In this review, we summarize current evidence on the role of neutrophils in the pathogenesis and progression of HCC and we highlight their potential utilization in HCC prognosis and therapy.

**Abstract:**

Hepatocellular carcinoma represents the most prevalent primary liver cancer worldwide, and it is either caused by intrinsic genetic mutations or by a multitude of extrinsic risk factors. Even though the interplay between chronic inflammatory changes and hepatocarcinogenesis has been at the forefront of clinical investigation for the past few decades, the role of tumor-associated neutrophils (TANs) in HCC development still remains ambiguous. On the one hand, N1 TANs exhibit an anti-tumorigenic activity, mediated by direct or indirect tumor cell lysis, whereas on the other hand, N2 TANs have been correlated with increased HCC growth, invasiveness, and metastasis. The association of an elevated Neutrophil-to-Lymphocyte Ratio (NLR) with poor prognosis in patients with HCC, has been recently brought into spotlight, consolidating its widespread use as a reliable biomarker. Due to the decisive involvement of TANs in HCC pathogenesis and development, the utilization of various neutrophil-centered anticancer treatment modalities has been under clinical experimentation, selectively targeting and modulating the processes of neutrophil recruitment, activation, and migration. This review summarizes current evidence on the role of TANs in HCC pathogenesis and progression, as well as in their potential involvement in tumor therapy, shedding light on emerging anticancer treatment methods targeting neutrophils.

## 1. Introduction

Liver cancer was the sixth most commonly diagnosed cancer and the fourth leading cause of cancer death worldwide in 2018, with about 841,000 new cases and 782,000 deaths annually [1,2]. Hepatocellular carcinoma is the most prevalent primary liver cancer, accounting for >80% of primary liver cancers worldwide, with geographical variations among its prevalence, and it is the leading cause of death in patients with cirrhosis, with an annual HCC incidence of 1–6% and patients with decompensated cirrhosis being at a particularly high risk [3]. Hepatocellular carcinogenesis includes angiogenesis, chronic inflammation, as well as alterations within the tumor macro- and micro-environment and is caused either by intrinsic factors that include inherited or acquired genetic mutations or by extrinsic risk factors such as chronic alcohol consumption, smoking, nonalcoholic fatty liver disease (NAFLD) and the hepatotropic viruses B, C, and D. HCC develops mostly on cirrhotic liver (about in 90% of the cases), whereas HCC development in normal liver is a rare event (less than 10% of the cases). It is widely accepted that all of the aforementioned factors can play a significant role in the development of HCC, by inducing alterations on mature hepatocytes or stem cells, leading to apoptosis, cell proliferation, dysplasia, and, eventually, neoplasia [4,5]. The diagnosis of HCC, relying solely on non-invasive criteria, is nowadays under debate due to the need for molecular information that necessitates tissue biopsies, while therapy is provided in accordance with tumor stages and the anticipated advantages of major interventions, following the Barcelona Clinic Liver Cancer (BCLC) staging system. Principally, resection, transplantation and local ablation are most commonly performed in patients with early-stage HCC tumors [6,7], while TACE and systemic therapy are the preferred treatment options for intermediate and advanced stage tumors, respectively [8,9].

Even though tumorigenesis involves cancer stem cells (CSCs), which are long-lived cells with increased self-renewal capacity [10], inflammation has come to light as the hallmark of cancer [11], while the interrelation between inflammatory changes and carcinogenesis has been well established for over 2000 years [12]. Chronic inflammation associated with cancer is known to alter the tumor microenvironment, via the infiltration of several immune cell populations into tumors, thus promoting HCC development [13]. Among others, it has become increasingly apparent over the past decade that neutrophils are implicated in the tumor-associated inflammation that drives disease progression and metastasis [14,15,16]. In the vast majority of solid human tumors, high infiltration with tumor-associated neutrophils (TANs) has been correlated with increased tumor growth, lymph node metastasis, and poor prognosis overall [17]. On the other hand, evidence from other studies advocate that under certain conditions, TANs exert direct or indirect cytotoxic and inhibitory activity towards tumor cells and cancer progression, respectively [18]. Furthermore, emerging data from pre-clinical studies evaluating various cancer therapeutic strategies targeting TANs have been recently brought into spotlight [19,20].

In this review, we summarize current evidence on the role of neutrophils in the pathogenesis and progression of HCC and we highlight their potential utilization in HCC prognosis and therapy.

## 2. The Dual Role of Myelopoiesis and Neutrophils in Cancer

**Figure 1:**
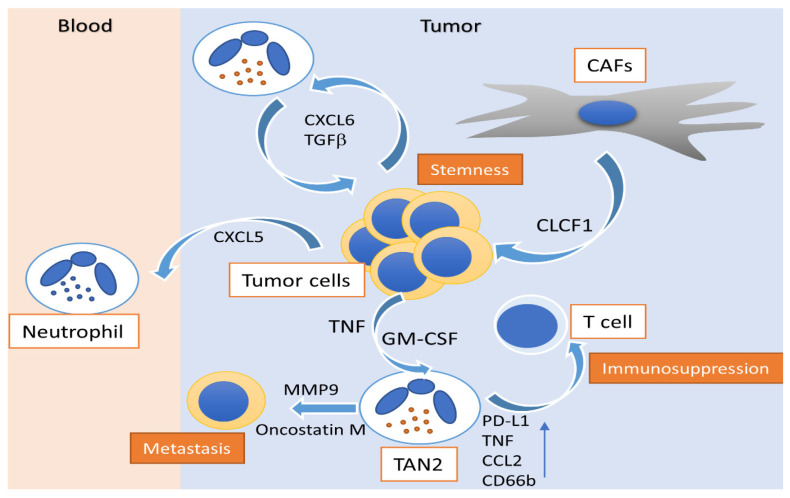
Activity of polarized N2 neutrophils in hepatocellular carcinoma. Peritumoral GM-CSF and CAFs modulate N2 neutrophils to an immunosuppressive profile, enhancing CD66b, PD-L1, TNF and CCL2 expression, alongside their capacity to suppress T cells. CAF-released CLCF1 drives the N2 polarization of TANs, induces CXCL6 and TGF-β production by tumor cells and promotes cancer stemness. N2 neutrophil-associated MMP-9 and oncostatin-M release promotes tumor neovascularization and enhances the metastatic potential of HCC. HCC: hepatocellular carcinoma; TAN: tumor-associated neutrophil; GM-CSF: granulocyte-macrophage colony-stimulating factor; TGFβ: tumor growth factor-β; TNF: tumor necrosis factor; PD-L1: programmed death-ligand 1; CXCL: C-X-C motif chemokine ligand; CCL2: C–C motif chemokine ligand 2; MMP-9: matrix metallopeptidase-9; CD66b: cluster of differentiation 66b; CAF: cancer-associated fibroblast; CLCF: cardiotrophin like cytokine factor.

Dysregulated myelopoiesis from hematopoietic stem and progenitors (HSPCs) to mature cells of granulopoietic and monocytic lineage is an important feature of cancer [21,22,23]. Tumor-associated inflammation is known to reprogram HSPCs towards the generation of myeloid cells with tumor promoting or suppressive properties [24]. Among other myeloid cell populations that are generated in the bone marrow, neutrophils, which are the first immune cells to be recruited following an injury or an infection [25], also infiltrate into tumors, where they acquire a specific immunosuppressive phenotype that supports tumor growth [26,27]. Infiltrating TANs exhibit either anti-tumorigenic (N1) or protumorigenic (N2) phenotypes [28]. For instance, low-density neutrophils (LDNs) and high-density neutrophils (HDNs) that were observed in a murine breast cancer model had diverse morphology and function. HDNs had a mature, segmented morphology, and exhibited antitumor phenotypes (N1). On the other hand, LDNs were a heterogeneous population of mature and immature (banded and ring-shaped) neutrophils and possessed protumor phenotypes (N2) (Figure 1).

Tumor-related inflammatory stimuli shape neutrophil phenotype in cancer. Transforming growth factor-beta TGF-β plays a major role in neutrophil plasticity, driving the acquisition of an N2 phenotype [29,30]. Moreover, the release of neutrophil extracellular traps (NETs), which are structures formed by DNA and granular proteins and are released upon stimulation, is linked to the N2 phenotype, as NETs contain proteins that support tumor growth, such as matrix metallopeptidase-9 (MMP-9) and cathepsin G [31,32,33,34]. Additionally, it has been advocated that NETs support metastasis by sequestering circulating tumor cells [35]. Christoffersson et al. also demonstrated that inflammatory neutrophils were recruited to sites of injury by C-X-C chemokine ligand (CXCL)2, whereas angiogenesis-promoting ones were recruited by vascular endothelial growth factor A (VEGF-A) [36].

**Figure 2:**
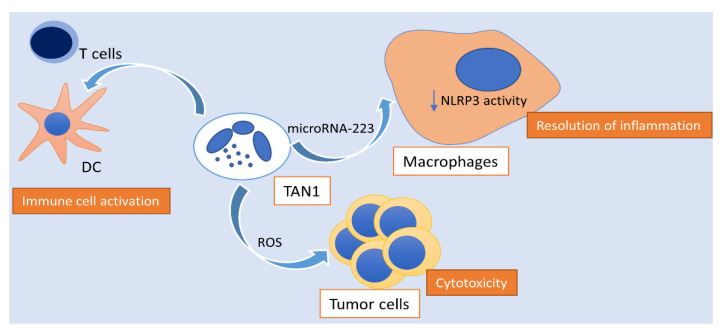
Activity of polarized N1 neutrophils in hepatocellular carcinoma. N1 TANs exhibit ROS-mediated cytotoxic activity towards tumor cells, activate the innate and adaptive immune response, as well as enhance the spontaneous resolution of tumor-associated inflammation via the transmission of microRNA-223 to macrophages, hindering NLRP3 activity. TAN: tumor-associated neutrophil; NLRP3: NLR family pyrin domain containing 3; ROS: reactive oxygen species; DC: dendritic cell.

On the other hand, type I interferons support the generation of TANs with antitumor properties (N1) and reactive oxygen species (ROS) were proven to be potent mediators of neutrophil antitumor activity, promoting direct tumor cell apoptosis (Figure 2). Using several ectopic and orthotopic mouse tumor models, Frindleder et al. demonstrated that blocking of the TGF-β signaling, accomplished via neutralization of the cytokine itself either via inhibition of its receptor (TGFβR) or via inhibition of the intracellular kinases involved in downstream signaling, resulted in recruitment and activation of CD8 + T cells, macrophages and a TAN phenotype with significant cytotoxic antitumor properties, characterized by the expression of CD101 and CD177 surface markers [37]. Along the same line, transcriptional and epigenetic reprogramming of neutrophils and their progenitors in the bone marrow upon induction of inflammation via β-glycan administration, resulted in N1 polarization of TANs, in a manner that was dependent on interferon-α signaling [38]. In fact, Sagiv et al. demonstrated that cancer-related HDNs had higher cytotoxicity towards tumor cells in culture and an increased oxidative burst compared to LDNs, which were associated with the expression of CD117, CD170, and programmed death ligand-1 (PD-L1) surface markers. Following a modified Winn assay test that assessed the implications of co-injecting tumor cells with either HDNs or LDNs upon tumor growth, data suggested that even though LDNs had no major effect on tumor growth, HDNs drastically hindered tumor growth demonstrating their antitumor properties [39]. Furthermore, Colombo et al., using modified adenocarcinoma cells that produced granulocyte-colony-stimulating factor (G-CSF), reached a conclusion that neutrophils are recruited by G-CSF into tumors, where they come in contact with tumor cells, inhibiting tumor development [40]. In addition, a study by Granot et al. showed that cancer-related HDNs were highly cytotoxic toward tumor cells in culture and that they dramatically reduced the seeding of disseminating tumor cells through the generation of ROS by the NAPDH oxidase complex [41]. Finally, a study by Matlung et al. demonstrated that neutrophils can exert a mode of destruction of cancer cells, involving antibody-mediated trogocytosis by neutrophils that can be further improved by targeting CD47-SIRPa interactions [42].

Recent studies have further showed that apart from neutrophils, their unipotent precursor cells (NeP) were mobilized in mouse melanoma models and in patients with tumor [43,44]. Increased numbers of these cells, bearing neutrophil and progenitor surface markers including CD66b, CD117, CD38, and CD71, were identified in the blood of patients with melanoma and in blood and tumors of patients with lung cancer [43]. These cells have immune suppressive and tumor promoting properties, as shown in a humanized mouse tumor model [44]. Considered together, neutrophil plasticity plays an important role in tumor pathophysiology, offering potential prognostic and therapeutic targets.

## 3. The Protumorigenic Role of Neutrophils in HCC

Multiple studies support the protumorigenic role of neutrophils in HCC (Table 1). A study by He et al. showed that granulocyte–macrophage colony-stimulating factor (GM-CSF) and tumor necrosis factor (TNF) are significantly expressed in the peritumoral area of HCC, modulating neutrophils to an immunosuppressive profile, enhancing the PD-L1 expression and their capacity to suppress T cells [45]. In addition, He et al. provided evidence that high infiltration of neutrophils in HCC determined malignant cell c-Met associated clinical outcome of patients and that peritumoral stromal neutrophils are essential for c-Met-elicited metastasis in human HCC [46]. Furthermore, recent evidence also emerged by Hsu et al. showcasing that pro-metastatic immature low-density neutrophils accumulated more efficiently in the livers of mice bearing metastatic lesions compared with anti-metastatic mature HDNs [47]. It has been also demonstrated that cancer-associated fibroblasts infiltrating HCC promote the activation and survival of TANs, which is reflected by an increased expression of CD66b, PD-L1, IL-8, TNF, and CCL2, but decreased expression of CD62L through an IL-6 signal transducer and activator of transcription-3 (STAT3)-PD-L1 signaling cascade [48]. Song et al. evaluated the interrelation among cancer associated fibroblasts (CAFs), HCC cells, and TANs, which is mediated via a network of cytokines. In a cohort of HCC clinical samples, the authors highlighted the role of the cardiotrophin-like cytokine factor 1 (CLCF1)-CXCL6/TGF-β axis in the regulation of cancer stemness, alongside the concomitant recruitment of “N2” TANs, contributing to the poor prognosis of HCC patients. Specifically, CLCF1 released by CAFs induced the production of CXCL6 and TGF-β by tumor cells, which acted on tumor cells themselves, promoting their stemness, and on TANs, driving their N2 polarization [49]. Zhou et al. have also advocated the existence of a positive feedback loop between cancer stem-like cells and TANs in HCC, controlling tumor progression and patient outcome. In the HCC microenvironment, TANs secrete bone morphogenetic protein 2 (BMP2), TGF-β2 and trigger miR-301b-3p expression in HCC cells, subsequently suppressing gene expression of limbic system–associated membrane protein (LSAMP), cylindromatosis lysine-63 deubiquitinase (CYLD) and as a consequence increase the stem cell-like characteristics of HCC cells. Those TAN-induced HCC stem-like cells were hyperactive in NF-κB signaling, secreting increased levels of CXCL5 while instigating TANs infiltration, suggesting a positive feedback loop [50]. Interestingly, another study by Peng et al. had similar results, showing that monocytes were educated by HCC microenvironment to upregulate their levels of glycolysis, which led to the production of large amounts of CXCL2 and CXCL8 via PFKFB3-NF-κB axis, recruiting peripheral neutrophils, potentially favoring tumor metastasis and facilitating disease progression of human HCC [51]. A recent study has shown that in non-alcoholic steatohepatitis (NASH), elevated free fatty acids stimulate the accumulation of NETs and promote the formation of HCC, suggesting an involvement of neutrophils in the development of HCC in patients with NASH [52]. It was also shown that infiltrating neutrophils promote angiogenesis in the tumor microenvironment acting as a primary source of MMP-9 [53].

Several studies suggest that the degree of TAN infiltration and the expression of TAN markers is correlated with disease prognosis. In a study, Zhou et al. suggested that Chemokine (C-X-C motif) ligand 5 (CXCL5) mediated the infiltration of neutrophils and subsequent HCC cell differentiation and invasion and that CXCL5 overexpression, alone or in combination with intratumoral neutrophil presence, acted as a prognostic indicator for overall survival (OS) [54]. Li et al. also found out that the increased presence of intratumoral CD66b+ neutrophils was highly associated with advanced Barcelona clinic liver cancer (BCLC) stage, liver fibrosis, elevated serum gamma-glutamyltransferase (γ-GT) and decreased recurrence-free survival and OS, and it was also considered a poor prognostic factor for HCC after resection [55].

Moreover, evidence surfaced demonstrating that CCL2 and CCL17 expression in TANs was correlated with tumor progression and prognosis in patients with HCC. The number of CCL2+ and CCL17+ TANs was found to be associated with tumor size, microvascular invasion, level of tumor differentiation, and staging. Those chemokines were preferably expressed by neutrophils throughout the tumor stroma but not by tumor cells or by the adjacent non-malignant tissues, while patients whose tumors had lower levels of CCL2+ or CCL17+ cells had longer survival times than those with higher numbers of these cells [56]. In addition, another study by Li et al. provided evidence that the majority of neutrophils in HCC intratumoral regions expressed autophagy specific protein LC3 and autophagosomes, while neutrophil autophagy was also correlated with sustained production of pro-metastatic oncostatin M and MMP9 and advanced migration of cancer cells [57]. Moreover, Kuang et al. provided direct evidence that the number of CD15+ neutrophils in the peritumoral stroma was significantly increased and was correlated with disease progression in HCC patients and their OS and that a high infiltration of peritumoral neutrophils was positively correlated with tumor size and angiogenesis progression at tumor-invading edge of HCC patients via MMP-9 signaling. They also demonstrated that neutrophil depletion effectively inhibited tumor angiogenesis and growth [58]. Consistent with the aforementioned findings, Wang et al. showed that intratumoral neutrophils infiltration was an independent prognostic factor for poor survival for HCC patients and that the percentage of intratumoral neutrophils infiltration was much higher in high indole-amine-2,3-dioxygenase (IDO) expression group than that in low IDO expression group, indicating that IDO might play a role in the recruitment of neutrophils [59]. In addition, the combination of CXCR2 and CXCL1 expression levels was proven to be a powerful predictor of poor prognosis for patients with HCC, as they regulate neutrophil infiltration into HCC tumor tissues [60].

**Table 1:** Summary of studies evaluating the role of TANs in HCC pathogenesis.

Study (year)	Study Subjects	Primary Outcome	Secondary Outcome
He et al. [45] (2015)	Human/Animal	Infiltration of neutrophils is markedly higher in peritumoral tissue than in the actual tumor site	TNF-α and GM-CSF increase PD-L1 expression on neutrophils in the TME
He et al. [46] (2016)	Human	HGF production by neutrophils is TME mediated	GM-CSF is required for tumor neutrophil activation and HGF production
Cheng et al. [48] (2018)	Human	CAFs regulate the survival, activation, and function of neutrophils within HCC through an IL6–STAT3–PDL1 signaling cascade	IL6 induces PDL1+ neutrophils via the JAK-STAT3 pathway, impairing T-cell function through PD1/PDL1 signaling
Song et al. [49] (2021)	Human/Animal	CLCF1/CXCL6/TGF-β axis upregulates the recruitment of “N2” TANs in HCC	Levels of CLCF1−CXCL6/TGF-β axis are correlated with the number of intratumoral “N2” TANs and HCC prognosis
Zhou et al. [50] (2019)	Human/Animal	TANs secrete BMP2, TGF-β2 and trigger miR-301b-3p expression in HCC cells, suppressing gene expression of LSAMP, CYLD and increasing HCC stemness	Increased TANs correlated with elevated miR-301b-3p, decreased LSAMP and CYLD expression, higher nuclear p65 accumulation and CXCL5 expression
Peng et al. [51] (2020)	Human	Monocyte derived CXCL2 and CXCL8 regulate the recruitment of neutrophils sustaining their accumulation and survival in the TME	Levels of PFKFB3, CXCL2/CXCL8 production in monocytes and infiltration of OSM-producing neutrophils are positively correlated in HCC
Van der Windt et al. [52] (2018)	Human/Animal	Neutrophils infiltrate murine NASH livers and undergo NET formation. NET inhibition reduces monocyte infiltration, inflammation and progression of NASH to HCC	Commonly elevated free fatty acids stimulate NET formation in vitro
Li et al. [57] (2015)	Human	Activation of ERK1/2, p38, and NF-κB is required for autophagy induction in tumor neutrophils	Increased neutrophil autophagy contributes to the mitochondrial stabilization-mediated cell survival and promotes metastasis
Calvente et al. [61] (2019)	Animal	Neutrophils contribute to spontaneous resolution of liver inflammation and fibrosis via miR-223	Neutrophils mediate the silencing of NLRP3 in proinflammatory macrophages via miR-223 and induce their alternative activation into a restorative phenotype after the cessation of injury

HCC: hepatocellular carcinoma; TAN: tumor-associated neutrophil; TNF: tumor necrosis factor; GM-CSF: granulocyte–macrophage colony-stimulating factor; PD-L1: programmed death ligand-1; TME: tumor microenvironment; ERK: extracellular signal-regulated kinase; NF: nuclear factor; HGF: hepatocyte growth factor; NET: neutrophil extracellular trap; NASH: nonalcoholic steatohepatitis; CAF: cancer-associated fibroblast; STAT: signal transducer and activator of transcription; IL: interleukin; JAK: janus kinase; LSAMP: limbic system-associated membrane protein; CYLD: CYLD lysine 63 deubiquitinase; NLRP3: NLR family pyrin domain containing 3; miR: microRNA; CXCL: chemokine C-X-C motif ligand; PFKFB: phosphofructo-2-kinase/fructose-2,6-biphosphatase; OSM: oncostatin-M; TGF-β: transforming growth factor-β; CLCF: cardiotrophin like cytokine factor; BMP: bone morphogenetic protein.

## 4. The Anti-Tumorigenic Role of Neutrophils in HCC

The anti-tumorigenic effect of HDN on tumor development and metastasis is mediated by direct cytotoxicity or by the activation of innate and adaptive immune cells, such as T and B lymphocytes, natural killer (NK), and dendritic cells (DCs) [62]. N1 TANs have been correlated with increased NADPH oxidase activity which leads to the formation of ROS, which are known to have cytotoxic properties to tumor cells [63,64]. More importantly, a recent in vivo study from Calvente et al. demonstrated by injecting Ly6G monoclonal antibodies in mice during recovery phases of two different chronic liver disease models, that neutrophils mediate the spontaneous resolution of liver inflammation and fibrosis via transmitting microRNA-223 to macrophages with the help of neutrophil-derived extracellular vesicles [61]. They found that neutrophil depletion impairs spontaneous resolution of liver inflammation and that neutrophil abrogation worsens spontaneous resolution of early liver fibrosis. These findings were correlated with an impairment of the phenotypic alteration of proinflammatory macrophages into a restorative stage after cessation of the stressor and an increased NLRP3/miR-223 ratio, suggesting that microRNA-223 is a key element that controls NLRP3 inflammasome proinflammatory activity in the liver. During inflammation, the expression of miR-223 is also upregulated through mechanisms that are incompletely understood yet, and the molecule binds to its complementary sequence in a regulatory part of NLRP3 mRNA (miR-223 target site in 3 ′UTR), leading to decreased NLRP3 mRNA and protein expression. Mice with deletion of the granulocyte-specific miR-223 gene exhibited an impaired resolution profile that could be overturned by replacing miR-223 levels using a miR-223 3p mimic or by infusion of neutrophils from wild type animals, suggesting that miR-223 might have a key role in neutrophil-associated resolution of inflammation after cessation of an exogenous stressor [65]. Along the same line, the pivotal role of miR-223, that is expressed at the highest levels in neutrophils, playing an important part in attenuating neutrophil maturation and activation, thereby suppressing liver inflammation and injury in several liver injury models [66,67,68], was brought to spotlight by He et al. [69]. Their study demonstrated that miR-223 ameliorates NASH and HCC by targeting multiple inflammatory and oncogenic genes, suggesting that overexpression of miR-223 could be a therapeutic strategy for the treatment of NASH and as a result, hinder the development and progression of HCC. Furthermore, Finisguerra et al. provided evidence that Met, a proto-oncogene, is required for the recruitment of antitumoral neutrophils and that Met deletion in neutrophils increased HCC growth, as it resulted in decreased nitric oxide (NO) dependent cancer cell killing [70].

## 5. Significance of Neutrophil-to-Lymphocyte Ratio in Hepatocellular Carcinoma as a Prognostic Marker

Various studies have provided strong evidence that an elevated Neutrophil-to-Lymphocyte Ratio (NLR) is associated with poor outcome for the patient, highlighting the importance of NLR as a reliable biomarker with prognostic potential for HCC (Table 2). First, He et al. showcased that a higher NLR was negatively associated with the OS of patients with HCC after surgery and that the patients with increased peritumoral CD66b+ neutrophils had decreased OS rate. Results demonstrated that intratumoral and peritumoral neutrophils had increased levels of PD-L1 expression compared to circulating neutrophils in patients with HCC, providing evidence that TANs had strong immunosuppressive effects in these patients and highlighting the potential role of PD-L1+ neutrophils as targets of anti-PD-1 and/or anti-PD-L1 antibodies in order to reverse their immunosuppressive phenotype [45]. Along the same line, in another study from Terashima et al. assessing the potential prognostic value of NLR of patients with advanced HCC, results showed that the median progression-free survival (PFS) and median OS of patients with high NLR were significantly shorter than that of patients with low NLR and that NLR was a predictor of response to hepatic arterial infusion chemotherapy (HAIC) in patients with advanced HCC [71]. In addition, a recent study from Kim et al. pointed out that hyperprogressive disease (HPD), which occurs in a subset of patients with HCC treated with PD-1 inhibitors, was associated with worse OS and PFS, and it could be predicted by an elevated (>4.125) NLR [72]. More importantly, a meta-analysis of 15 individual studies by Xiao et al. concluded that elevated NLR was associated with poor OS and disease-free survival in HCC initially treated with liver transplantation and surgical resection, as well as in HCC treated by radiofrequency ablation. Moreover, high NLR was highly correlated with the presence of vascular invasion, tumor multifocality and higher incidence of AFP ≥ 400 ng/mL [73]. Finally, another meta-analysis of 13 individual studies by Xu et al., reached a conclusion that an elevated pre-transplant NLR had a close association with the OS and disease-free survival of patients undergoing liver transplantation for HCC, as well as with the presence of vascular invasion and Milan criteria for liver transplantation [74]. Based on current evidence however, no definitive predictive factors/scores are useful for the prediction of the outcome of advanced HCC patients, in which the role of neutrophils is more important compared to other therapeutic settings [75].

## 6. The Role of Neutrophils in Response to Systemic Treatment of HCC

In recent years, major scientific breakthroughs have broadened our knowledge regarding the pathogenesis of HCC. Emerging data from whole exome and genome sequencing have brought into spotlight the main signaling pathways that are altered in HCC (telomere, cell cycle, Wnt/b-catenin, epigenetic, NRF2/KEAP1, RAS/RAF/MAPK, AKT/mTOR pathways), enabling us to better understand the link between genetic and transcriptomic alterations, as well as clinical and pathological features that define homogenous subgroups of HCC [77]. Moreover, several integrative molecular classifications have been published in addition to prognostic molecular signatures deriving from cancerous and non-cancerous liver [78]. Recently, scientists have also described the main genetic and transcriptomic data on HCC, further potentiating the translation of genomic knowledge into clinical practice [7].

Different combinations of immune checkpoint inhibitors (ICIs) targeting the programmed cell death-1 (PD-1) pathway and the application of antiangiogenic therapies are at the forefront of combination therapy trials for patients with HCC. Evidence provided by Finn et al. showed that the use of atezolizumab in combination with bevacizumab reduced mortality by 42% and decreased the risk of disease worsening or death by 41% compared to sorafenib alone. It is also of note that more than 20% of patients under the combination therapy had a stable PFS for more than 13 months, while PFS was nearly zero for patients under the sole use of sorafenib [79]. Along the same line, Zhou et al. showed that sorafenib treatment increased TAN infiltration in animal models and HCC patients by inducing CXCL5 expression in HCC cells via the hypoxic HIF1α-NF-kB pathway, while experimentally, TAN depletion inhibited tumor growth and angiogenesis to a greater extent when combined with sorafenib [56].

It would be of major interest, however, to further exploit the antitumor potential of neutrophils, either by targeting them directly or their microenvironment, in patients with HCC. In fact, experimentation with different approach methods could take place. First, different stimuli instigate the production of hydrogen peroxide (H_2_O_2_) and an oxidative burst in neutrophils. Blocking signaling through TGFβR enhances the production of H_2_O_2_, triggering an intracellular signaling pathway in tumor cells, leading to the activation of the non-selective transient receptor potential cation channel subfamily M member 2 (TRPM2), inducing a lethal influx of calcium into tumor cells [80]. Additionally, the expression of HGF by neutrophils, which acts on the HGF receptor (HGFR; also known as MET), promotes nitric oxide synthase (iNOS) expression, inducing the release of NO, resulting in tumor cell lysis [70]. By blocking RTK, which is the receptor of HGF on tumor cells, the HGF in HCC microenvironment could act as the stimulator of NO by TANs. Moreover, inhibition of the CXC-chemokine receptor-1 (CXCR1) or of the CXCR2, halts the propagation of immunosuppressive neutrophils in cancer. In addition, neutrophil reprogramming by blocking of the TGFβR, IFN-β signaling or antagonism of angiotensin II type 1 receptor (AGTR1), can enhance the anti-tumorigenic activity of neutrophils, while CXCR4 blockade promotes IL-18 production by neutrophils and ΝΚ-cell activation [81,82].

## 7. Anticancer Treatment Options Targeting Neutrophils

Neutrophils can be also cellular targets of anticancer therapy, mainly via altering the processes of neutrophil recruitment, migration, or activation (Table 3). There is strong evidence that the degradation of tumor-associated macrophages via antibodies targeting colony stimulating factor 1 (CSF1), leads to a compensatory activation of neutrophils, which is mediated by carcinoma-associated fibroblasts. To overcome this hurdle, combined inhibition of neutrophils alongside macrophages was successfully utilized, significantly reducing tumor growth, demonstrating increased treatment efficiency [83]. Moreover, inhibition of neutrophil infiltration by anti-granulocyte receptor 1 depletion or combined CXCR2-formyl peptide receptor 1 antagonism significantly reduced liver injury in mice [84]. Along the same line, blockade of the CXCL-1 or of the intercellular adhesion molecule-1 expression decreased hepatic neutrophil infiltration and diminished liver injury and fibrosis [85]. Additionally, priming of TANs with cytokines resulted in a shift of the polarization of neutrophils, potentiating their tumor suppressing effect [86]. Note, however, that increased serum interleukin-8 (IL-8), which recruits neutrophils while stimulating angiogenesis and tumor cell proliferation, was correlated with enhanced intra-tumor neutrophils and decreased clinical benefit of immune checkpoint inhibitors (ICIs), highlighting the significance of evaluating serum IL-8 levels in identifying unfavorable tumor immunobiology and as an independent biomarker in patients receiving ICIs. Moreover, evidence surfaced that IL-8 is associated with poor prognosis for patients with cancer treated with ICIs, reporting that IL-8 is produced by myeloid cells both in the tumor and blood (neutrophils and monocytes), correlating with an immunosuppressive myeloid-enriched tumor microenvironment with reduced IFNG RNA signatures and T cell effector activity [87,88,89]. Those studies demonstrate that IL-8 has a significant role in determining the types and quantity of myeloid cell infiltrating tumors, providing more evidence for utilizing inhibitors of the IL-8 axis agents, that either inhibit IL-8 itself or its receptors (CXCR1/CXCR2), for anticancer treatment, either as monotherapy or in combination with other agents. A recent study by Haider et al. provided evidence that the synergy of TGF-β and Axl induces CXCL5 secretion, causing the infiltration of neutrophils into HCC tissue, proposing that the intervention with TGF-β/Axl/CXCL5 signaling would be an effective therapeutic strategy to combat HCC progression in TGF-β-positive patients [90]. The expression of the immunoglobulin G (IgG) and IgA Fc receptors by neutrophils, results into the destruction of antibody-opsonized cancer cells through the process of antibody-dependent cellular cytotoxicity (ADCC) [91]. Interestingly, IgA-elicited neutrophil-mediated ADCC can be amplified by simultaneous blocking of the CD47-signal regulatory protein-α (SIRPα) myeloid checkpoint [92].

Furthermore, Imai et al. provided evidence that the increased invasive activity of tumor cells co-cultured with neutrophils in patients with HCC, was significantly suppressed by the addition of anti-hepatocyte growth factor antibody [93]. Note that the release of TNF-a into the circulation during recombinant IL-2 therapy of cancer patients is often associated with marked activation of granulocytes that cause lysis of tumor cells, mediating most of the therapeutic efficacy [94,95]. Additionally, donor-derived neutrophils from healthy subjects have exerted tumor cell killing capabilities upon recipients [96]. More importantly, trained immunity mediated by β-glycan, resulted in an epigenetic shift of granulopoiesis towards the production of neutrophils with antitumor properties, potentiated by increased ROS formation. The beneficial effect of “trained” neutrophils was established by the suppressed tumor growth in mice that received β-glycan donor-derived neutrophils [97]. It has been also demonstrated that neutrophils, monocytes and macrophages express SIRPα, acting as a phagocytosis checkpoint via its interaction with the trogoptosis inhibition signal CD47 that is presented on target cells. CD47 is overexpressed on cancer cells, increasing their resistance to myeloid cells [98]. Thus, CD47–SIRPα checkpoint blockade would potentially increase the destruction of tumor cells, through the process of trogoptosis. Furthermore, a recent study by Yang et al. provided evidence that NET formation was enhanced in neutrophils derived from HCC patients, inducing cell death resistance and enhanced invasiveness, which was mediated by internalization of NETs into trapped HCC cells and activation of Toll-like receptors TLR4/9-COX2 signaling. Inhibition of TLR4/9-COX2 signaling revoked the NET-induced metastatic potential, while a combination of DNase 1 directly destroying NETs with anti-inflammatory drugs aspirin/hydroxychloroquine significantly reduced HCC metastasis in mice model [76]. Note that lenzilumab, a humaneered anti-human GM-CSF monoclonal antibody that directly binds GM-CSF and prevents signaling through its receptor, may prove to be potentially useful clinically in the future as an inhibitor of GM-CSF mediated recruitment of N2 TANs and macrophages in the TME of HCC [99]. Finally, via the application of the novel CIBERSORT method, which allows immune cell profiling by deconvolution of gene expression microarray data, Rohr-Udilova et al. reported important data about the innate and adaptive immune cell composition of patients with HCC. Results showed that increased total immune cell infiltration into HCC correlated with total B cells, memory B cells, T follicular helper cells, and M1 macrophages, whereas weak infiltration was linked to resting NK cells, neutrophils, and resting mast cells [100].

**Table 3:** Summary of studies evaluating the role of TANs in HCC therapy.

Study (year)	Study Subjects	Outcome
Zhou et al. [56] (2016)	Human/Animal	The combination of sorafenib and TAN depletion inhibits tumor growth and neovascularization to a greater extent than sorafenib alone
Calvente et al. [61] (2019)	Animal	Neutrophils contribute to spontaneous resolution of liver inflammation and fibrosis via microRNA-223 mediated activation of restorative macrophages that release IL-10
He et al. [67] (2017)	Animal	Deletion of the ICAM-1 gene ameliorates neutrophil infiltration and liver injury in miR-223 knockout mice
He et al. [69] (2019)	Human/Animal	Neutrophil-expressed miR-223 hinders the progression of HCC by targeting multiple inflammatory and oncogenic genes
Finisguerra et al. [70] (2015)	Human/Animal	Administration of MET kinase inhibitor counters the therapeutic benefit of MET targeting in cancer cells by the pro-tumoral effect arising from MET blockade in neutrophils
Marques et al. [84] (2012)	Human/Animal	Blockage of neutrophil infiltration by GR-1 depletion or combined CXCR2-FPR1 antagonism significantly prevents hepatotoxicity
Zhou et al. [85] (2018)	Animal	Blockade of CXCL1 or ICAM-1 expression reduces hepatic neutrophil infiltration and ameliorates liver injury and fibrosis
Imai et al. [93] (2005)	Human	The increased invasive activity of tumor cells co-cultured with neutrophils in patients with HCC is significantly suppressed by the addition of anti-HGF antibody
Yang et al. [76] (2020)	Human/Animal	Inhibition of TLR4/9-COX2 signaling abrogates the NET-aroused metastatic potential of HCC

TAN: tumor-associated neutrophil; HCC: hepatocellular carcinoma; HGF: hepatocyte growth factor; GR-1: anti-granulocyte receptor-1; CXCR: C-X-C motif chemokine receptor; FPR: formyl peptide receptor; MET: hepatocyte growth factor receptor; ICAM: intercellular adhesion molecule; miR: microRNA; CXCL: chemokine C-X-C motif ligand; TLR: toll-like receptor; IL: interleukin; COX: cyclooxygenase; NET: neutrophil extracellular trap.

## 8. Critical Analysis of Data and Future Perspectives

HCC tumor immune microenvironment (TME) affects response to current anti–PD-1/PD-L1 immunotherapies. Therefore, an enhanced understanding of the immunobiology of TME is essential for the development of predictive biomarkers of patient stratification and strategies of drug combinations to improve therapeutic efficacy, especially for patients with tumors irresponsive to anti–PD-1/PD-L1 therapy. A tumor-suppressive immune ecosystem is characterized by the presence of neutrophils with a TAN N1 phenotype with significant cytotoxic anti-tumor properties, characterized as CD101+, CD177+, CD170low, CD54+, HLA-DR+, CD86+, and CD15high, whereas a tumor-progressive immune ecosystem is characterized by the presence of neutrophils with an immunosuppressive N2 polarization of TANs, characterized as CD170high and PD-L1+. Note that, at least in vitro, neutrophils usually make fewer molecules of a given cytokine than monocytes/macrophages or lymphocytes do on a per-cell basis [101]. In vivo, however, neutrophils constitute the majority of infiltrating cells in inflamed tissues, which may be also true for cancer TME, often outnumbering mononuclear leukocytes by one to two orders of magnitude. Thus, the fact that neutrophils clearly predominate over other cell types under various in vivo conditions is indicative that, under those circumstances, the contribution of neutrophil-derived cytokines and chemokines to HCC addiction loops (Figure 1) can be decisive. It would be of major interest to further exploit the antitumor potential of neutrophils and their products, either by targeting them directly, or their microenvironment, in patients with HCC.

The majority of reviews about tumor-progressive immune TME in HCC, focus on immunosuppressive myeloid-derived suppressor cell (MDSC)-like macrophages, tumor-associated macrophage (TAM)-like macrophages (with CD86, CD206, CD163, HLA-DR surface markers), LAMP3+ dendritic cells (DCs), layilin (LAYN)+ regulatory T cells (Tregs), and dysfunctional LAYN+ CD8 T cells. Herein, we reviewed focused experimental and clinical evidence regarding the existence of several HCC promoting, mechanistically acting TAN derived cytokines, chemokines, molecules and TME cell to cell loop interactions, that are amenable to potential interventions that increase treatment efficacy (Table 4). Due to complicated nature of myeloid inflammation, multiple target inhibition might be needed in order to overcome the myeloid-mediated immune suppression. In this context, both VEGF-A and IL-8 contribute to angiogenesis and myeloid inflammation and are concomitantly highly expressed in tumor-infiltrating myeloid cells. The potential development of IL-8 pathway-blocking agents alongside ICIs, might improve the treatment efficacy in certain tumors as well as in the tumor immunogenic context.

## 9. Conclusions

In conclusion, controversial data support that under certain conditions, neutrophils can act either as the orchestrators of HCC development or as the cells counteracting its propagation. Moreover, evidence from translational studies highlight the importance of targeting the tumor microenvironment in order to overcome resistance to current treatment modalities, while the potential use of neutrophils and NLR as prognostic indicators of tumor progression is advocated by many studies and is of major clinical importance, alongside the involvement of neutrophils in novel therapeutic strategies targeting HCC. Single-cell analysis is the new method that has enabled us to better understand inter- as well as intratumoral heterogeneity and has contributed to the identification of new potential targets for immunotherapy or multimodal treatments. However, unravelling the diversity of TANs at the single-cell level and translating that knowledge to neutrophil function, as well as the use of gene-targeting approach methods for neutrophil depletion and inhibition of select neutrophil functions, represent important future challenges in the field. Taking into account the health burden of HCC worldwide, enhancing our knowledge of innovative prognostic methods and experimentation with different treatment modalities, should be of utmost priority in our seemingly never-ending fight against hepatocellular cancer.

## Figures and Tables

**Table 2:** Summary of studies evaluating the role of TANs in HCC prognosis.

Study (year)	Study Subjects	Primary Outcome	Secondary Outcome
He et al. [45] (2015)	Human/Animal	High NLR is negatively correlated with the OS of patients with HCC	The ratio of PD-L1+ neutrophils-to-PD-1+ T cells is higher in peritumoral tissue and better predicts the DFS of patients with HCC
Zhou et al. [54] (2012)	Human/Animal	CXCL5 overexpression alone, or combined with the presence of intratumoral neutrophils, is an independent prognostic indicator for OS and cumulative recurrence	CXCL5 promotes HCC cell proliferation, invasion, and intratumoral neutrophil infiltration via the activation of the PI3K-Akt and ERK1/2 signaling pathways
Li et al. [55] (2011)	Human	Increased intratumoral neutrophils are significantly associated with decreased RFS/OS and are identified as an independent prognostic factor of patients with HCC	Intratumoral CD66b+ neutrophils significantly correlated with CD8+ T cells, TGF-β expression, BCLC stage and early recurrence of HCC, whereas peritumoral neutrophils were not associated with the outcome of HCC
Zhou et al. [56] (2016)	Human/Animal	The number of CCL2+ or CCL17+ TANs correlates with tumor size, microvascular invasion, tumor encapsulation, tumor differentiation, and stage	Patients whose tumors have lower levels of CCL2+ or CCL17+ cells have longer survival times than those with higher numbers of these cells.
Kuang et al. [58] (2011)	Human	Accumulation of neutrophils in peritumoral stroma fosters disease progression via MMP-9 and predicts reduced survival in HCC patients	Accumulation of peritumoral stromal neutrophils coincides with increased VEGF expression and angiogenesis progression at the invading tumor edge of HCC
Wang et al. [59] (2019)	Human	Both IDO expression and intratumoral neutrophils infiltration were independent prognostic factors for OS after resection	High IDO expression is a risk factor for intratumoral neutrophils infiltration in HCC patients
Terashima et al. [71] (2015)	Human	The objective response rate to HAIC of advanced HCC patients with low NLR is significantly better than that of patients with high NLR	Median PFS and median OS in patients with high NLR is significantly shorter than that of the patients with low NLR
Kim et al. [72] (2021)	Human	Elevated NLR explicitly predicts the occurrence of HPD as well as inferior survival rate after PD-1 blockade	HPD is associated with worse PFS and OS as well as deprivation of chances for subsequent treatments
Xiao et al. [73] (2014)	Human	High NLR is associated with poor OS and DFS in HCC initially treated by liver transplantation and surgical resection and significantly correlates with the presence of vascular invasion tumor multifocality and higher incidence of AFP ≥ 400 ng/mL	High NLR is associated with poor OS in HCC treated by radiofrequency-ablation, TACE and mixed treatment consisting of locoregional, systemic treatments or supportive care
Xu et al. [74] (2018)	Human	Elevated pretransplant NLR has a close association with the OS, RFS and DFS of patients undergoing LT for HCC	Elevated NLR is associated with the presence of vascular invasion and Milan criteria
Yang et al. [76] (2020)	Human/Animal	NET formation is enhanced in neutrophils from patients with HCC, especially metastatic HCC	NETs enhance metastatic potential of the trapped HCC cells through activating TLR4/9

TAN: tumor-associated neutrophil; HCC: hepatocellular carcinoma; MMP-9: matrix metallopeptidase-9; VEGF: vascular endothelial growth factor; RFS: recurrence-free survival; OS: overall survival; DFS: disease-free survival; NLR: neutrophil-to-lymphocyte ratio; AFP: alpha-fetoprotein; HAIC: hepatic arterial infusion chemotherapy; CCL: C-C motif chemokine ligand; LT: liver transplantation; IDO: indoleamine 2,3-dioxygenase; HPD: hyperprogressive disease; PD-1: Programmed death-1; NET: neutrophil extracellular trap; TLR: toll-like receptor; CXCL: C-X-C motif chemokine ligand; TGF-β: transforming growth factor-β; BCLC: Barcelona Clinic Liver Cancer; VEGF: vascular endothelial growth factor; TACE: transarterial chemoembolization; ERK: extracellular signal-regulated kinase; PI3K: phosphoinositide 3-kinase; Akt: protein kinase B.

**Table 4:** Summary of potential therapeutic interventions and targets of TANs in HCC that may increase treatment efficacy in future trials.

Therapeutic Intervention		Targeted Pro-Tumorigenic Activity in HCC
**TGF-β/TGFβR/SMADs inhibition**	anti-TGF-β monoclonal antibodies; Lerde-, Mete-, Freso-limumab TGFβR I inhibitor; LY364947 TGF-β inhibitors; trabedersen, lucanix TGFβR I/II kinase inhibitors; i.e., LY2157299	TGF-β high local concentrations “educate” N2 TANs TGF-β/Axl/CXCL5 signaling CAFs/TANs—CLCF1-CXCL6/TGF-β axis CSCs/TANs-BMP2-TGF-β2-miR-301b-3p/CXCL5
**JAK2 inhibition**	Tofacitinib, Baricitinib, Upadacitinib	CAFs/TANs—IL8-CCL2-IL6/JAK2/STAT3-PD-L1 axis
**Interferon administration**	interferon beta-1b (Betaferon)	IFN-β positive signaling polarization of N1 TANs
**IL8 inhibition**	HuMax-IL8 (BMS-986253); anti-IL-8 monoclonal antibody	CXCR1/CXCR2/CXCL-1/IL8 recruitment of N2 TANs
**CXCL5/CXCR2 inhibition**	Reparixin; non-competitive CXCR1/2 antagonist, SCH- 527123; CXCR2 antagonist, LY294002; PI3K inhibitor	HCC-HIF1α-NF-kB/CXCL5/CXCR2-TAN axis
**GM-CSF inhibition**	Lenzilumab; anti-human GM-CSF monoclonal antibody	GM-CSF mediated recruitment of N2 TANs

HCC: hepatocellular carcinoma; JAK: janus kinase; TGF-β: transforming growth factor beta; TGFβR: transforming growth factor beta receptor; TAN: tumor-associated neutrophil; CAF: cancer associated fibroblast; CSC: cancer stem cell; CXCL5: C-X-C motif chemokine 5; CXCR2: C-X-C motif chemokine receptor 2; GM-CSF: granulocyte-macrophage colony-stimulating factor; PI3K: phosphoinositide 3-kinase.

## Data Availability

No new data were created or analyzed in this study. Data sharing is not applicable to this article.

## References

[1] Bray F., Ferlay J., Soerjomataram I., Siegel R.L., Torre L.A., Jemal A. (2018). Global cancer statistics 2018: GLOBOCAN estimates of incidence and mortality worldwide for 36 cancers in 185 countries. CA Cancer J. Clin..

[2] Ferlay J., Colombet M., Soerjomataram I., Mathers C., Parkin D.M., Pineros M., Znaor A., Bray F. (2019). Estimating the global cancer incidence and mortality in 2018: GLOBOCAN sources and methods. Int. J. Cancer.

[3] Akinyemiju T., Abera S., Ahmed M., Alam N., Alemayohu M.A., Allen C., Al-Raddadi R., Alvis-Guzman N., Amoako Y., Global Burden of Disease Liver Cancer Collaboration (2017). The Burden of Primary Liver Cancer and Underlying Etiologies From 1990 to 2015 at the Global, Regional, and National Level: Results from the Global Burden of Disease Study 2015. JAMA Oncol..

[4] Ghouri Y.A., Mian I., Rowe J.H. (2017). Review of hepatocellular carcinoma: Epidemiology, etiology, and carcinogenesis. J. Carcinog..

[5] Thorgeirsson S.S., Grisham J.W. (2002). Molecular pathogenesis of human hepatocellular carcinoma. Nat. Genet..

[6] Centonze L., Di Sandro S., Lauterio A., De Carlis R., Frassoni S., Rampoldi A., Tuscano B., Bagnardi V., Vanzulli A., De Carlis L. (2021). Surgical Resection vs. Percutaneous Ablation for Single Hepatocellular Carcinoma: Exploring the Impact of Li-RADS Classification on Oncological Outcomes. Cancers.

[7] Llovet J.M., Kelley R.K., Villanueva A., Singal A.G., Pikarsky E., Roayaie S., Lencioni R., Koike K., Zucman-Rossi J., Finn R.S. (2021). Hepatocellular carcinoma. Nat. Rev. Dis. Primers.

[8] Llovet J.M., Villanueva A., Marrero J.A., Schwartz M., Meyer T., Galle P.R., Lencioni R., Greten T.F., Kudo M., Mandrekar S.J. (2021). Trial Design and Endpoints in Hepatocellular Carcinoma: AASLD Consensus Conference. Hepatology.

[9] European Association for the Study of the Liver (2018). EASL Clinical Practice Guidelines: Management of hepatocellular carcinoma. J. Hepatol..

[10] Nguyen L.V., Vanner R., Dirks P., Eaves C.J. (2012). Cancer stem cells: An evolving concept. Nat. Rev. Cancer.

[11] Mantovani A. (2009). Cancer: Inflaming metastasis. Nature.

[12] Balkwill F., Mantovani A. (2001). Inflammation and cancer: Back to Virchow?. Lancet.

[13] Grivennikov S.I., Greten F.R., Karin M. (2010). Immunity, inflammation, and cancer. Cell.

[14] Coffelt S.B., Wellenstein M.D., de Visser K.E. (2016). Neutrophils in cancer: Neutral no more. Nat. Rev. Cancer.

[15] Galdiero M.R., Varricchi G., Loffredo S., Mantovani A., Marone G. (2018). Roles of neutrophils in cancer growth and progression. J. Leukoc. Biol..

[16] Makarova-Rusher O.V., Medina-Echeverz J., Duffy A.G., Greten T.F. (2015). The yin and yang of evasion and immune activation in HCC. J. Hepatol..

[17] Shen M., Hu P., Donskov F., Wang G., Liu Q., Du J. (2014). Tumor-associated neutrophils as a new prognostic factor in cancer: A systematic review and meta-analysis. PLoS ONE.

[18] Gerrard T.L., Cohen D.J., Kaplan A.M. (1981). Human neutrophil-mediated cytotoxicity to tumor cells. J. Natl. Cancer Inst..

[19] Gregory A.D., Houghton A.M. (2011). Tumor-associated neutrophils: New targets for cancer therapy. Cancer Res..

[20] Quail D.F., Joyce J.A. (2013). Microenvironmental regulation of tumor progression and metastasis. Nat. Med..

[21] McAllister S.S., Weinberg R.A. (2014). The tumour-induced systemic environment as a critical regulator of cancer progression and metastasis. Nat. Cell Biol..

[22] Shojaei F., Wu X., Zhong C., Yu L., Liang X.H., Yao J., Blanchard D., Bais C., Peale F.V., van Bruggen N. (2007). Bv8 regulates myeloid-cell-dependent tumour angiogenesis. Nature.

[23] Hattori K., Heissig B., Tashiro K., Honjo T., Tateno M., Shieh J.H., Hackett N.R., Quitoriano M.S., Crystal R.G., Rafii S. (2001). Plasma elevation of stromal cell-derived factor-1 induces mobilization of mature and immature hematopoietic progenitor and stem cells. Blood.

[24] Marvel D., Gabrilovich D.I. (2015). Myeloid-derived suppressor cells in the tumor microenvironment: Expect the unexpected. J. Clin. Investig..

[25] Sionov R.V., Fridlender Z.G., Granot Z. (2015). The Multifaceted Roles Neutrophils Play in the Tumor Microenvironment. Cancer Microenviron..

[26] Uribe-Querol E., Rosales C. (2015). Neutrophils in Cancer: Two Sides of the Same Coin. J. Immunol. Res..

[27] Patel S., Fu S., Mastio J., Dominguez G.A., Purohit A., Kossenkov A., Lin C., Alicea-Torres K., Sehgal M., Nefedova Y. (2018). Unique pattern of neutrophil migration and function during tumor progression. Nat. Immunol..

[28] Kalafati L., Mitroulis I., Verginis P., Chavakis T., Kourtzelis I. (2020). Neutrophils as Orchestrators in Tumor Development and Metastasis Formation. Front. Oncol..

[29] Fridlender Z.G., Albelda S.M. (2012). Tumor-associated neutrophils: Friend or foe?. Carcinogenesis.

[30] Piccard H., Muschel R.J., Opdenakker G. (2012). On the dual roles and polarized phenotypes of neutrophils in tumor development and progression. Crit. Rev. Oncol. Hematol..

[31] Wilson T.J., Nannuru K.C., Futakuchi M., Sadanandam A., Singh R.K. (2008). Cathepsin G enhances mammary tumor-induced osteolysis by generating soluble receptor activator of nuclear factor-kappaB ligand. Cancer Res..

[32] Boeltz S., Amini P., Anders H.J., Andrade F., Bilyy R., Chatfield S., Cichon I., Clancy D.M., Desai J., Dumych T. (2019). To NET or not to NET:current opinions and state of the science regarding the formation of neutrophil extracellular traps. Cell Death Differ..

[33] Cools-Lartigue J., Spicer J., Najmeh S., Ferri L. (2014). Neutrophil extracellular traps in cancer progression. Cell Mol. Life Sci..

[34] Albrengues J., Shields M.A., Ng D., Park C.G., Ambrico A., Poindexter M.E., Upadhyay P., Uyeminami D.L., Pommier A., Kuttner V. (2018). Neutrophil extracellular traps produced during inflammation awaken dormant cancer cells in mice. Science.

[35] Cools-Lartigue J., Spicer J., McDonald B., Gowing S., Chow S., Giannias B., Bourdeau F., Kubes P., Ferri L. (2013). Neutrophil extracellular traps sequester circulating tumor cells and promote metastasis. J. Clin. Investig..

[36] Christoffersson G., Vagesjo E., Vandooren J., Liden M., Massena S., Reinert R.B., Brissova M., Powers A.C., Opdenakker G., Phillipson M. (2012). VEGF-A recruits a proangiogenic MMP-9-delivering neutrophil subset that induces angiogenesis in transplanted hypoxic tissue. Blood.

[37] Fridlender Z.G., Sun J., Kim S., Kapoor V., Cheng G., Ling L., Worthen G.S., Albelda S.M. (2009). Polarization of tumor-associated neutrophil phenotype by TGF-beta: “N1” versus “N2” TAN. Cancer Cell.

[38] Andzinski L., Kasnitz N., Stahnke S., Wu C.F., Gereke M., von Kockritz-Blickwede M., Schilling B., Brandau S., Weiss S., Jablonska J. (2016). Type I IFNs induce anti-tumor polarization of tumor associated neutrophils in mice and human. Int. J. Cancer.

[39] Sagiv J.Y., Michaeli J., Assi S., Mishalian I., Kisos H., Levy L., Damti P., Lumbroso D., Polyansky L., Sionov R.V. (2015). Phenotypic diversity and plasticity in circulating neutrophil subpopulations in cancer. Cell Rep..

[40] Colombo M.P., Lombardi L., Stoppacciaro A., Melani C., Parenza M., Bottazzi B., Parmiani G. (1992). Granulocyte colony-stimulating factor (G-CSF) gene transduction in murine adenocarcinoma drives neutrophil-mediated tumor inhibition in vivo. Neutrophils discriminate between G-CSF-producing and G-CSF-nonproducing tumor cells. J. Immunol..

[41] Granot Z., Henke E., Comen E.A., King T.A., Norton L., Benezra R. (2011). Tumor entrained neutrophils inhibit seeding in the premetastatic lung. Cancer Cell.

[42] Matlung H.L., Babes L., Zhao X.W., van Houdt M., Treffers L.W., van Rees D.J., Franke K., Schornagel K., Verkuijlen P., Janssen H. (2018). Neutrophils Kill Antibody-Opsonized Cancer Cells by Trogoptosis. Cell Rep..

[43] Dinh H.Q., Eggert T., Meyer M.A., Zhu Y.P., Olingy C.E., Llewellyn R., Wu R., Hedrick C.C. (2020). Coexpression of CD71 and CD117 Identifies an Early Unipotent Neutrophil Progenitor Population in Human Bone Marrow. Immunity.

[44] Zhu Y.P., Padgett L., Dinh H.Q., Marcovecchio P., Blatchley A., Wu R., Ehinger E., Kim C., Mikulski Z., Seumois G. (2018). Identification of an Early Unipotent Neutrophil Progenitor with Pro-tumoral Activity in Mouse and Human Bone Marrow. Cell Rep..

[45] He G., Zhang H., Zhou J., Wang B., Chen Y., Kong Y., Xie X., Wang X., Fei R., Wei L. (2015). Peritumoural neutrophils negatively regulate adaptive immunity via the PD-L1/PD-1 signalling pathway in hepatocellular carcinoma. J. Exp. Clin. Cancer Res..

[46] He M., Peng A., Huang X.Z., Shi D.C., Wang J.C., Zhao Q., Lin H., Kuang D.M., Ke P.F., Lao X.M. (2016). Peritumoral stromal neutrophils are essential for c-Met-elicited metastasis in human hepatocellular carcinoma. Oncoimmunology.

[47] Hsu B.E., Roy J., Mouhanna J., Rayes R.F., Ramsay L., Tabaries S., Annis M.G., Watson I.R., Spicer J.D., Costantino S. (2020). C3a elicits unique migratory responses in immature low-density neutrophils. Oncogene.

[48] Cheng Y., Li H., Deng Y., Tai Y., Zeng K., Zhang Y., Liu W., Zhang Q., Yang Y. (2018). Cancer-associated fibroblasts induce PDL1+ neutrophils through the IL6-STAT3 pathway that foster immune suppression in hepatocellular carcinoma. Cell Death Dis..

[49] Song M., He J., Pan Q.Z., Yang J., Zhao J., Zhang Y.J., Huang Y., Tang Y., Wang Q., He J. (2021). Cancer-associated fibroblast-mediated cellular crosstalk supports hepatocellular carcinoma progression. Hepatology.

[50] Zhou S.L., Yin D., Hu Z.Q., Luo C.B., Zhou Z.J., Xin H.Y., Yang X.R., Shi Y.H., Wang Z., Huang X.W. (2019). A Positive Feedback Loop Between Cancer Stem-Like Cells and Tumor-Associated Neutrophils Controls Hepatocellular Carcinoma Progression. Hepatology.

[51] Peng Z.P., Jiang Z.Z., Guo H.F., Zhou M.M., Huang Y.F., Ning W.R., Huang J.H., Zheng L., Wu Y. (2020). Glycolytic activation of monocytes regulates the accumulation and function of neutrophils in human hepatocellular carcinoma. J. Hepatol..

[52] van der Windt D.J., Sud V., Zhang H., Varley P.R., Goswami J., Yazdani H.O., Tohme S., Loughran P., O’Doherty R.M., Minervini M.I. (2018). Neutrophil extracellular traps promote inflammation and development of hepatocellular carcinoma in nonalcoholic steatohepatitis. Hepatology.

[53] Ardi V.C., Kupriyanova T.A., Deryugina E.I., Quigley J.P. (2007). Human neutrophils uniquely release TIMP-free MMP-9 to provide a potent catalytic stimulator of angiogenesis. Proc. Natl. Acad. Sci. USA.

[54] Zhou S.L., Dai Z., Zhou Z.J., Wang X.Y., Yang G.H., Wang Z., Huang X.W., Fan J., Zhou J. (2012). Overexpression of CXCL5 mediates neutrophil infiltration and indicates poor prognosis for hepatocellular carcinoma. Hepatology.

[55] Li Y.W., Qiu S.J., Fan J., Zhou J., Gao Q., Xiao Y.S., Xu Y.F. (2011). Intratumoral neutrophils: A poor prognostic factor for hepatocellular carcinoma following resection. J. Hepatol..

[56] Zhou S.L., Zhou Z.J., Hu Z.Q., Huang X.W., Wang Z., Chen E.B., Fan J., Cao Y., Dai Z., Zhou J. (2016). Tumor-Associated Neutrophils Recruit Macrophages and T-Regulatory Cells to Promote Progression of Hepatocellular Carcinoma and Resistance to Sorafenib. Gastroenterology.

[57] Li X.F., Chen D.P., Ouyang F.Z., Chen M.M., Wu Y., Kuang D.M., Zheng L. (2015). Increased autophagy sustains the survival and pro-tumourigenic effects of neutrophils in human hepatocellular carcinoma. J. Hepatol..

[58] Kuang D.M., Zhao Q., Wu Y., Peng C., Wang J., Xu Z., Yin X.Y., Zheng L. (2011). Peritumoral neutrophils link inflammatory response to disease progression by fostering angiogenesis in hepatocellular carcinoma. J. Hepatol..

[59] Wang Y., Yao R., Zhang L., Xie X., Chen R., Ren Z. (2019). IDO and intra-tumoral neutrophils were independent prognostic factors for overall survival for hepatocellular carcinoma. J. Clin. Lab. Anal..

[60] Li L., Xu L., Yan J., Zhen Z.J., Ji Y., Liu C.Q., Lau W.Y., Zheng L., Xu J. (2015). CXCR2-CXCL1 axis is correlated with neutrophil infiltration and predicts a poor prognosis in hepatocellular carcinoma. J. Exp. Clin. Cancer Res..

[61] Calvente C.J., Tameda M., Johnson C.D., Del Pilar H., Lin Y.C., Adronikou N., De Mollerat Du Jeu X., Llorente C., Boyer J., Feldstein A.E. (2019). Neutrophils contribute to spontaneous resolution of liver inflammation and fibrosis via microRNA-223. J. Clin. Invest..

[62] Jaillon S., Galdiero M.R., Del Prete D., Cassatella M.A., Garlanda C., Mantovani A. (2013). Neutrophils in innate and adaptive immunity. Semin. Immunopathol..

[63] Jaillon S., Ponzetta A., Di Mitri D., Santoni A., Bonecchi R., Mantovani A. (2020). Neutrophil diversity and plasticity in tumour progression and therapy. Nat. Rev. Cancer.

[64] Eun H.S., Chun K., Song I.S., Oh C.H., Seong I.O., Yeo M.K., Kim K.H. (2019). High nuclear NADPH oxidase 4 expression levels are correlated with cancer development and poor prognosis in hepatocellular carcinoma. Pathology.

[65] Knorr J., Wree A., Tacke F., Feldstein A.E. (2020). The NLRP3 Inflammasome in Alcoholic and Nonalcoholic Steatohepatitis. Semin. Liver Dis..

[66] Ye D., Zhang T., Lou G., Liu Y. (2018). Role of miR-223 in the pathophysiology of liver diseases. Exp. Mol. Med..

[67] He Y., Feng D., Li M., Gao Y., Ramirez T., Cao H., Kim S.J., Yang Y., Cai Y., Ju C. (2017). Hepatic mitochondrial DNA/Toll-like receptor 9/MicroRNA-223 forms a negative feedback loop to limit neutrophil overactivation and acetaminophen hepatotoxicity in mice. Hepatology.

[68] Li M., He Y., Zhou Z., Ramirez T., Gao Y., Gao Y., Ross R.A., Cao H., Cai Y., Xu M. (2017). MicroRNA-223 ameliorates alcoholic liver injury by inhibiting the IL-6-p47(phox)-oxidative stress pathway in neutrophils. Gut.

[69] He Y., Hwang S., Cai Y., Kim S.J., Xu M., Yang D., Guillot A., Feng D., Seo W., Hou X. (2019). MicroRNA-223 Ameliorates Nonalcoholic Steatohepatitis and Cancer by Targeting Multiple Inflammatory and Oncogenic Genes in Hepatocytes. Hepatology.

[70] Finisguerra V., Di Conza G., Di Matteo M., Serneels J., Costa S., Thompson A.A., Wauters E., Walmsley S., Prenen H., Granot Z. (2015). MET is required for the recruitment of anti-tumoural neutrophils. Nature.

[71] Terashima T., Yamashita T., Iida N., Yamashita T., Nakagawa H., Arai K., Kitamura K., Kagaya T., Sakai Y., Mizukoshi E. (2015). Blood neutrophil to lymphocyte ratio as a predictor in patients with advanced hepatocellular carcinoma treated with hepatic arterial infusion chemotherapy. Hepatol. Res..

[72] Kim C.G., Kim C., Yoon S.E., Kim K.H., Choi S.J., Kang B., Kim H.R., Park S.H., Shin E.C., Kim Y.Y. (2021). Hyperprogressive disease during PD-1 blockade in patients with advanced hepatocellular carcinoma. J. Hepatol..

[73] Xiao W.K., Chen D., Li S.Q., Fu S.J., Peng B.G., Liang L.J. (2014). Prognostic significance of neutrophil-lymphocyte ratio in hepatocellular carcinoma: A meta-analysis. BMC Cancer.

[74] Xu Z.G., Ye C.J., Liu L.X., Wu G., Zhao Z.X., Wang Y.Z., Shi B.Q., Wang Y.H. (2018). The pretransplant neutrophil-lymphocyte ratio as a new prognostic predictor after liver transplantation for hepatocellular cancer: A systematic review and meta-analysis. Biomark Med..

[75] Marasco G., Colecchia A., Bacchi Reggiani M.L., Celsa C., Farinati F., Giannini E.G., Benevento F., Rapaccini G.L., Caturelli E., Di Marco M. (2020). Comparison of prognostic models in advanced hepatocellular carcinoma patients undergoing Sorafenib: A multicenter study. Dig. Liver Dis..

[76] Yang L.Y., Luo Q., Lu L., Zhu W.W., Sun H.T., Wei R., Lin Z.F., Wang X.Y., Wang C.Q., Lu M. (2020). Increased neutrophil extracellular traps promote metastasis potential of hepatocellular carcinoma via provoking tumorous inflammatory response. J. Hematol. Oncol..

[77] Niu Z.S., Niu X.J., Wang W.H. (2016). Genetic alterations in hepatocellular carcinoma: An update. World J. Gastroenterol..

[78] Erstad D.J., Fuchs B.C., Tanabe K.K. (2018). Molecular signatures in hepatocellular carcinoma: A step toward rationally designed cancer therapy. Cancer.

[79] Finn R.S., Qin S., Ikeda M., Galle P.R., Ducreux M., Kim T.Y., Kudo M., Breder V., Merle P., Kaseb A.O. (2020). Atezolizumab plus Bevacizumab in Unresectable Hepatocellular Carcinoma. N. Engl. J. Med..

[80] Gershkovitz M., Caspi Y., Fainsod-Levi T., Katz B., Michaeli J., Khawaled S., Lev S., Polyansky L., Shaul M.E., Sionov R.V. (2018). TRPM2 Mediates Neutrophil Killing of Disseminated Tumor Cells. Cancer Res..

[81] Shrestha S., Noh J.M., Kim S.Y., Ham H.Y., Kim Y.J., Yun Y.J., Kim M.J., Kwon M.S., Song D.K., Hong C.W. (2016). Angiotensin converting enzyme inhibitors and angiotensin II receptor antagonist attenuate tumor growth via polarization of neutrophils toward an antitumor phenotype. Oncoimmunology.

[82] Yang J., Kumar A., Vilgelm A.E., Chen S.C., Ayers G.D., Novitskiy S.V., Joyce S., Richmond A. (2018). Loss of CXCR4 in Myeloid Cells Enhances Antitumor Immunity and Reduces Melanoma Growth through NK Cell and FASL Mechanisms. Cancer Immunol. Res..

[83] Kumar V., Donthireddy L., Marvel D., Condamine T., Wang F., Lavilla-Alonso S., Hashimoto A., Vonteddu P., Behera R., Goins M.A. (2017). Cancer-Associated Fibroblasts Neutralize the Anti-tumor Effect of CSF1 Receptor Blockade by Inducing PMN-MDSC Infiltration of Tumors. Cancer Cell.

[84] Marques P.E., Amaral S.S., Pires D.A., Nogueira L.L., Soriani F.M., Lima B.H., Lopes G.A., Russo R.C., Avila T.V., Melgaco J.G. (2012). Chemokines and mitochondrial products activate neutrophils to amplify organ injury during mouse acute liver failure. Hepatology.

[85] Zhou Z., Xu M.J., Cai Y., Wang W., Jiang J.X., Varga Z.V., Feng D., Pacher P., Kunos G., Torok N.J. (2018). Neutrophil-Hepatic Stellate Cell Interactions Promote Fibrosis in Experimental Steatohepatitis. Cell Mol. Gastroenterol. Hepatol..

[86] Sun R., Luo J., Li D., Shu Y., Luo C., Wang S.S., Qin J., Zhang G.M., Feng Z.H. (2014). Neutrophils with protumor potential could efficiently suppress tumor growth after cytokine priming and in presence of normal NK cells. Oncotarget.

[87] Bakouny Z., Choueiri T.K. (2020). IL-8 and cancer prognosis on immunotherapy. Nat. Med..

[88] Schalper K.A., Carleton M., Zhou M., Chen T., Feng Y., Huang S.P., Walsh A.M., Baxi V., Pandya D., Baradet T. (2020). Elevated serum interleukin-8 is associated with enhanced intratumor neutrophils and reduced clinical benefit of immune-checkpoint inhibitors. Nat. Med..

[89] Yuen K.C., Liu L.F., Gupta V., Madireddi S., Keerthivasan S., Li C., Rishipathak D., Williams P., Kadel E.E., Koeppen H. (2020). High systemic and tumor-associated IL-8 correlates with reduced clinical benefit of PD-L1 blockade. Nat. Med..

[90] Haider C., Hnat J., Wagner R., Huber H., Timelthaler G., Grubinger M., Coulouarn C., Schreiner W., Schlangen K., Sieghart W. (2019). Transforming Growth Factor-beta and Axl Induce CXCL5 and Neutrophil Recruitment in Hepatocellular Carcinoma. Hepatology.

[91] Brandsma A.M., Bondza S., Evers M., Koutstaal R., Nederend M., Jansen J.H.M., Rosner T., Valerius T., Leusen J.H.W., Ten Broeke T. (2019). Potent Fc Receptor Signaling by IgA Leads to Superior Killing of Cancer Cells by Neutrophils Compared to IgG. Front. Immunol..

[92] Treffers L.W., Ten Broeke T., Rosner T., Jansen J.H.M., van Houdt M., Kahle S., Schornagel K., Verkuijlen P., Prins J.M., Franke K. (2020). IgA-Mediated Killing of Tumor Cells by Neutrophils Is Enhanced by CD47-SIRPalpha Checkpoint Inhibition. Cancer Immunol. Res..

[93] Imai Y., Kubota Y., Yamamoto S., Tsuji K., Shimatani M., Shibatani N., Takamido S., Matsushita M., Okazaki K. (2005). Neutrophils enhance invasion activity of human cholangiocellular carcinoma and hepatocellular carcinoma cells: An in vitro study. J. Gastroenterol. Hepatol..

[94] Gemlo B.T., Palladino M.A., Jaffe H.S., Espevik T.P., Rayner A.A. (1988). Circulating cytokines in patients with metastatic cancer treated with recombinant interleukin 2 and lymphokine-activated killer cells. Cancer Res..

[95] Blay J.Y., Favrot M.C., Negrier S., Combaret V., Chouaib S., Mercatello A., Kaemmerlen P., Franks C.R., Philip T. (1990). Correlation between clinical response to interleukin 2 therapy and sustained production of tumor necrosis factor. Cancer Res..

[96] Yan J., Kloecker G., Fleming C., Bousamra M., Hansen R., Hu X., Ding C., Cai Y., Xiang D., Donninger H. (2014). Human polymorphonuclear neutrophils specifically recognize and kill cancerous cells. Oncoimmunology.

[97] Kalafati L., Kourtzelis I., Schulte-Schrepping J., Li X., Hatzioannou A., Grinenko T., Hagag E., Sinha A., Has C., Dietz S. (2020). Innate Immune Training of Granulopoiesis Promotes Anti-tumor Activity. Cell.

[98] Casey S.C., Tong L., Li Y., Do R., Walz S., Fitzgerald K.N., Gouw A.M., Baylot V., Gutgemann I., Eilers M. (2016). MYC regulates the antitumor immune response through CD47 and PD-L1. Science.

[99] Tan-Garcia A., Lai F., Sheng Yeong J.P., Irac S.E., Ng P.Y., Msallam R., Tatt Lim J.C., Wai L.E., Tham C.Y.L., Choo S.P. (2020). Liver fibrosis and CD206(+) macrophage accumulation are suppressed by anti-GM-CSF therapy. JHEP Rep..

[100] Rohr-Udilova N., Klinglmuller F., Schulte-Hermann R., Stift J., Herac M., Salzmann M., Finotello F., Timelthaler G., Oberhuber G., Pinter M. (2018). Deviations of the immune cell landscape between healthy liver and hepatocellular carcinoma. Sci. Rep..

[101] Tecchio C., Cassatella M.A. (2016). Neutrophil-derived chemokines on the road to immunity. Semin. Immunol..

